# Quality Improvement Project to Improve the Resident Doctors’ Out-of-Hours Clinical Handover System at Nottinghamshire Healthcare NHS Foundation Trust (NHFT)

**DOI:** 10.1192/bjo.2025.10331

**Published:** 2025-06-20

**Authors:** Ayodeji Akerele, Abiodun Omonori, Deepa Bagepalli Krishnan, Catherine Faruqi, Kehinde Junaid

**Affiliations:** Nottinghamshire Healthcare NHS Foundation Trust, Nottingham, United Kingdom

## Abstract

**Aims:** An effective and safe clinical handover system is at the heart of safe healthcare delivery, ensuring continuity of care between clinical teams. Handovers are completed face-to-face or verbally and recorded within the NHFT’s SharePoint handover system, the standard agreed upon within the Trust. This project aimed to improve the usability, access, and safety of a preexisting SharePoint handover system.

**Methods:** A pre-implementation survey with 30 responses from the Resident doctors showed that 90% of respondents were aware of the handover system. Still, only 60% carried out face-to-face handovers regularly, while 40% relied on other methods. 35% viewed the SharePoint handover system positively, but 50% found it inefficient, suggesting improvements. Model for Improvement Quality Improvement Methodology was used to design and develop this change project; working alongside key stakeholders (Resident doctors, Medical Education unit, Quality Improvement team and Information Technology (IT) professionals), changes were made using a Plan-Do-Study-Act (PDSA) framework to improve awareness, access, usability and accuracy of the SharePoint handover system. Awareness improved through sessions in the Resident doctors’ induction, emails and medical education newsletter. Working in collaboration with the IT team, the SharePoint system was securely moved to a safe server with changes made to the template and dropdown options to improve safety and accuracy. Automatic email reminders were set up to improve handover job completion and recording. A PowerBI dashboard was created to assess system use and the quality of the handover recording to ensure ongoing quality assurance and improvements.

**Results:** Six-week baseline data showed that the compliance rate of handovers was 80%, with 20% of handovers indicating neither face-to-face nor verbal communication. Only 20% (42 out of 209) of the jobs were marked complete, against standards of 100%.

After implementing change ideas, four-week data showed 100% compliance, indicating that all handovers were completed and recorded. Only 23.03% of the jobs were marked complete on the handover system, indicating an area for further improvement.

**Conclusion:** A Trustwide Standard Operating Procedure for Resident Doctor Handover is being developed, and further IT changes are planned to continuously monitor and improve the handover system. In this case, collaborative leadership, perseverance when encountering roadblocks, and a systematic data-driven improvement approach with iterative changes helped establish a safer, more usable, and accessible handover system.

